# Breast cancer stem cells tolerate chromosomal instability during tumor progression via c-Jun/AXL stress signaling

**DOI:** 10.1016/j.heliyon.2023.e20182

**Published:** 2023-09-14

**Authors:** Shahnawaz A. Baba, Qi Sun, Samson Mugisha, Shreyas Labhsetwar, Richard Klemke, Jay S. Desgrosellier

**Affiliations:** a Department of Pathology, University of California, San Diego, La Jolla, CA 92093, USA; b Moores Cancer Center, University of California,San Diego, La Jolla, CA 92093, USA

**Keywords:** Breast cancer, Stem cell activation, Chromosomal missegregation, Stress tolerance, Tumor initiation

## Abstract

Chromosomal instability (CIN) is critical for tumor evolution, yet its relationship with stemness is unclear. Here, we describe CIN as a key stress induced during tumor initiation that is uniquely tolerated by breast cancer stem cells in an activated signaling state (aCSCs). While we noted elevated CIN specifically in tumors from aCSCs, this was not intrinsic to these cells, as baseline levels were similar to non-stem cell types. This suggests that CIN is induced during tumor initiation, and that aCSCs can better tolerate this stress. Further, this increased CIN may be transient, as it was only in low-burden aCSC tumors, with levels diminishing in more established disease. Phospho-array profiling revealed specific activation of c-Jun stress signaling in aCSCs, which we hypothesized could induce genes responsible for CIN tolerance. Indeed, we identified *AXL* as a c-Jun dependent gene enriched in aCSCs that enhances resistance to this stress. Thus, CIN tolerance mediated by c-Jun/AXL signaling may be a defining feature of stemness, contributing to breast cancer progression.

## Highlights

• CIN is a critical stress induced during early tumor initiation.
• aCSCs uniquely tolerate high CIN levels in tumors.
• Basal CIN levels do not distinguish stem from non-stem cancer cells.
• Enhanced activation of c-Jun/AXL stress signaling is a unique feature of aCSCs.
• AXL mediates CIN tolerance in aCSCs.

## 1 Introduction

Tumor-initiating cancer stem cells (CSCs) sharing properties with adult mammary stem cells (MaSCs) are critical for breast cancer progression and metastasis [[1], [2], [3], [4]]. An underappreciated aspect of adult MaSCs biology is that they are highly dynamic, undergoing frequent changes in cell state due to hormonal signaling. In fact, stem cells activated by hormones are key drivers of mammary gland epithelial remodeling and branching morphogenesis during the menstrual cycle and pregnancy. Our prior studies showed that the cell surface receptor integrin αvβ3 is a critical marker of the activated stem cell state in the mammary gland during pregnancy [5]. In breast cancer, we further characterized αvβ3 as a marker of an aggressive population of stem-like cancer cells [6] similar to MaSCs activated by hormones in the normal human mammary gland [7]. Based on this similarity, we now refer to these cells as activated CSCs (aCSCs). These cells represent a subset of those identified by traditional CSC markers [6], and predict eventual metastasis in patients independent of tumor subtype [6], highlighting their aggressive nature. Together, our findings emphasize the need to identify critical vulnerabilities in aCSCs that could represent new targets for therapy.

Toward this goal, we recently identified a unique stress tolerance pathway that protects aCSCs against chromosomal instability (CIN) [7], a key endogenous stress linked to metastasis [8]. CIN is defined by continuous chromosomal missegregation, which causes significant stress in cancer cells, reducing cellular fitness. Thus, the ability to resist this stress may be an important feature of aggressive breast cancer cells. In fact, our unbiased analysis of critical genes expressed by aCSCs led to the surprising discovery of a TGFBI (BIG-H3)-ZEB1 signaling module that promotes stemness by decreasing CIN [7]. Consistent with our findings, normal MaSCs also use ZEB1 to reduce CIN during oncogenic transformation [9]. Together, this indicates that proper regulation of CIN may be critical for stemness. Paradoxically, recent studies also showed that high-CIN was required for metastasis in some breast cancer cells [8]. This further highlights the need to determine how CIN levels compare in stem and non-stem cell types and the role this plays in aggressive disease.

While CIN is a defining hallmark of cancer, little is known about its relationship with stemness. Furthermore, recent studies showed that high-CIN was required for metastasis [8], suggesting that it may be a unique feature of the most aggressive breast cancer cells. Thus, in this study we set out to characterize an association between CIN levels and aCSCs. To address this, we measured CIN in stem and non-stem cell types, and examined any changes that occurred during tumor initiation. Since CIN is a critical stress leading to decreased cancer cell survival, we also hypothesized that aCSCs might possess unique mechanisms allowing them to counteract these negative effects. Thus, we also sought to identify new signaling pathways in aCSCs that could promote CIN tolerance.

## 2 Materials and methods

### 2.1 Cell lines

Breast cancer cell lines purchased from ATCC (Manassas, VA, USA) include: HCC38, MCF-7, T47D, BT474, MDA-MB-468, BT-20, HCC1187, Hs578T, BT549, and MDA-MB-231. LM2-4 cells, a highly metastatic variant of the MDA-MB-231 cell line [10], was a gift from Robert Kerbel. All cell lines were shown to be free of mycoplasma. The identity of cell lines was confirmed by short tandem repeat (STR) testing. Cells used in mice were additionally tested and found to be negative for an extensive panel of mouse pathogens. Cell lines were cultured in complete DMEM medium (DMEM supplemented with 10% fetal bovine serum (FBS) + 1% l-glutamine, sodium pyruvate, non-essential amino acids, and antibiotic/antimycotic).

### 2.2 Anaphase missegregation counts

Orthotopic breast tumors in mice were generated from sorted HCC38 cells and harvested after 14 weeks as previously described [7]. For H&E-stained formalin-fixed paraffin-embedded tumor sections, CIN was quantified by manually counting anaphase cells. For cultured cell lines, DAPI fluorescent nuclear staining was performed in a 4-well chamberslide (Nunc, Thermo Fisher Scientific). Cells were fixed briefly in 2% paraformaldehyde in PBS at RT and permeabilized, followed by incubation with DAPI for 10 min. Stained cells were then imaged with a Leica DMi8 automated inverted microscope and anaphase cells manually counted under a 40x objective.

### 2.3 Lentivirus production and generation of CRISPR knockout cells

Plasmids containing enhanced specificity Cas9 and guide RNA's in the pLentiCRISPRv2 vector were purchased from GenScript (Piscataway, NJ, USA). Transient transfections for all CRISPR/Cas9 vectors into 293T cells were performed with Lipofectamine 3000 (Invitrogen, Thermo Fisher Scientific, Waltham, MA, USA) according to the manufacturer's instructions. Stable deletion of *AXL* was achieved by transducing LM2-4 cells with lentivirus expressing enhanced specificity Cas9 and guide RNA (GenScript), and pooling puromycin-resistant cells. A vector lacking the guide RNA was used as a negative control. Successful *AXL* deletion was verified by Western blot.

### 2.4 Bioinformatics analysis

The public gene expression profile from breast cancer cell lines [11] was obtained at NCBI GEO (GSE50470) and the corresponding intrinsic subtype information obtained from Prat et al. [12]. The CIN signature scores were calculated by comparing the gene set defined by Bakhoum et al. [8] with each cell line according to Barbie et al. [13], implemented in the gseapy python package (v0.9.8).

### 2.5 Real-time qPCR

qPCR experiments were performed by collecting total RNA from cultured cells with the RNeasy Mini Kit (Qiagen) and reverse transcribing with the High-Capacity cDNA Reverse Transcription Kit (Applied Biosystems, Thermo Fisher Scientific). For JNK inhibitor studies, cells were treated with vehicle (DMSO) or the indicated doses of SP600125 or JNK–IN–8 (Selleckchem, Houston, TX, USA) for 24 h prior to harvesting RNA. HCC38 cells were sorted as previously described [7] and relative mRNA levels examined from 90,000 cells using the Cells-to-CT kit (Life Technologies) according to manufacturer's instructions. Real-time qPCR was performed using iTaq Universal SYBR Green Supermix (Bio-Rad, Hercules, CA, USA) and run on a LightCycler 480 qPCR System (Roche, Basel, Switzerland). *AXL* primer sequences (5′-3′): forward – GTGGGCAACCCAGGGAATATC and reverse – GTACTGTCCCGTGTCGGAAAG.

### 2.6 Phospho-arrays and immunoblotting

Human phospho-kinase array kits (ARY003B, R&D Systems, Minneapolis, MN, USA) were blotted as per manufacturer's protocol. HCC38 cells were sorted as previously described [7]. Whole cell lysates were prepared with RIPA lysis buffer (100 mM Tris pH 7.5, 150 mM sodium chloride, 0.1% deoxycholate, 0.1% SDS, 50 mM NaF, Protease inhibitor cocktail (Roche), 2 mM PMSF, 2 mM sodium orthovanadate) combined with scraping and the lysates cleared by centrifugation. Standard Western blotting procedures were performed. The following primary antibodies were used for immunoblotting at a dilution of 1:1000: AXL (8661, Cell Signaling Technology, Danvers, MA, USA), c-Jun (2315, Cell Signaling Technology), pS63 c-Jun (91,952, Cell Signaling Technology), Hsp90 (sc-13119, Santa Cruz, Dallas, TX, USA), or 1:4000 β-actin (MABT825, MilliporeSigma). Treatments with 5 μM JNK–IN–8 (Selleckchem) or vehicle control (DMSO) were performed for 24 h prior to harvesting lysates. All blots or gels derive from the same experiment and were processed in parallel. See Supplementary Material for unedited blots (Supplementary Figs. S5 and S6).

### 2.7 Tumorsphere assays

Primary tumorsphere formation in methylcellulose was assessed in cells grown under anchorage-independent conditions. LM2-4 cells (10,000) were cultured in 0.9 mL of 1% methylcellulose/complete DMEM medium in ultra-low adhesion 24-well dishes (Corning, Corning, NY, USA) and cells cultured for 8–10 days. Cells were treated with vehicle (DMSO) or the indicated doses of SP600125, JNK–IN–8, or Reversine (all from Selleckchem) concurrent with embedding in methylcellulose. Primary tumorspheres were assessed by counting colonies consisting of at least 6 cells from 4 fields per well with a 10x objective.

### 2.8 Cell viability

XTT cell viability assays were performed by first seeding 4000 LM2-4 cells into a 96-well tissue culture plate. After cells attached overnight the indicated concentrations of Reversine (Selleckchem) or vehicle (DMSO) were then added to the wells in 100 μL phenol-free complete DMEM medium. After 48 h, XTT substrate (Thermo Fisher Scientific) was added to the wells and incubated for 2 h before reading the A450 nm on a plate reader.

### 2.9 Statistics

Data presentation and statistical tests are indicated in the figure legends. Two-tailed Student's t-tests were used for comparing two means while ANOVA was performed for 3 or more data sets. Post-hoc analysis was performed using appropriate multiple comparisons tests as indicated in the legends. For all analyses, *P* < 0.05 was considered statistically significant. Statistical analysis was performed using GraphPad Prism software (San Diego, CA, USA).

## 3 Results

### 3.1 High-CIN is associated with low-burden aCSC tumors

To examine potential differences in CIN levels between stem and non-stem cell types, we selected the HCC38 cell line. These cells more accurately reflect the cellular heterogeneity present in patients’ tumors compared to other cell lines [11], including a population of aCSCs with an EpCAM^Low^/αvβ3^+^ surface marker profile. Our prior studies showed these aCSCs are enriched for stemness properties such as tumorsphere formation and self-renewal [6] as well as *in vivo* tumor initiation [7]. Despite harvesting these animals at the same timepoint, we observed significant differences in primary tumor burden. We exploited this to further investigate how CIN might change during tumor progression, with low-burden tumors (<100 mm^3^) representing earlier stages of initiation and large tumors (>100 mm^3^) representing more established disease.

**Fig. 1:**
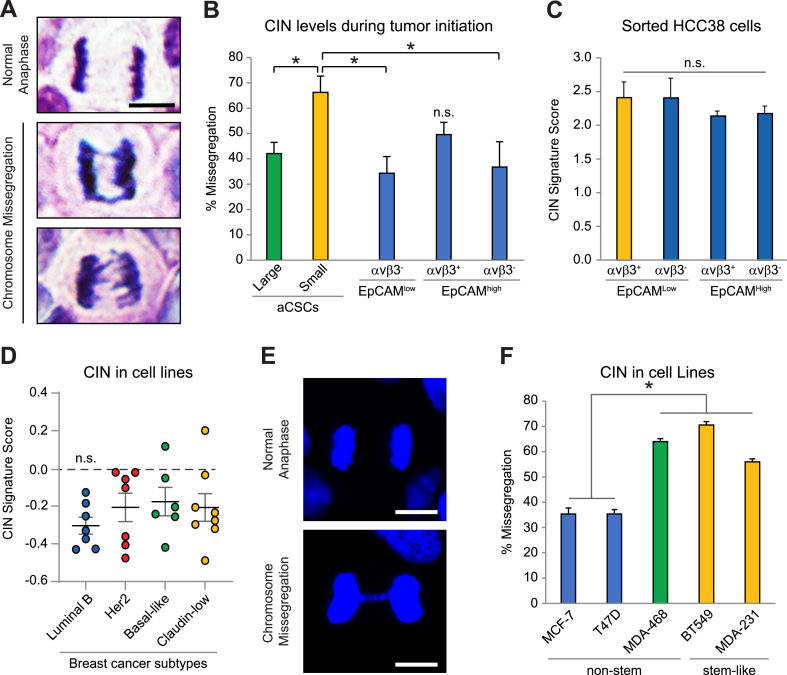
**CIN is a bottleneck to tumor initiation uniquely tolerated by aCSCs.** (**A**) Representative H&E images of anaphase cells in tumor xenografts initiated by aCSCs. Examples are displayed of normal anaphase (top) and chromosomal missegregations including anaphase bridges (middle) and lagging strands (bottom). Scale bar, 5 μm. (**B**) Quantitation of the missegregation frequency in tumors formed from limiting numbers of sorted HCC38 cells. Data from aCSCs (EpCAM^Low^/αvβ3^+^) was separated into small (<100 mm3) versus large tumors (>100 mm3). *P*-values relative to Small aCSC tumors (n = 3): Large aCSC (n = 7), *P* = 0.0379; EpCAM^Low^/αvβ3^-^ (n = 4), *P* = 0.0255; EpCAM^High^/αvβ3^+^ (n = 6), *P* = 0.102; EpCAM^High^/αvβ3^-^ (n = 4), *P* = 0.0325. >50 total anaphase cells assessed per tumor type. *P < 0.05. n. s. = not significant. (**C** and **D**) CIN gene signature scores for freshly sorted HCC38 cells from culture (**C**) or breast cancer cell lines representing different molecular subtypes (**D**). (**D**) Center line represents the mean with each dot indicating a different cell line. Cells in each category: Luminal B; n = 7, HER2; n = 7, Basal-like; n = 6, Claudin-low; n = 8. (**E** and **F**) Assessment of chromosomal missegregations by DAPI DNA staining. (**E**) Representative images of LM2-4 cancer cells in normal anaphase (top) or with an anaphase bridge (bottom). Scale bar, 10 μm. (**F**) Frequency of missegregations in breast cancer cell lines. *P < 0.05 for MCF-7 and T47D versus MDA-MB-468, BT549 and MDA-MB-231 cells. For each cell line, 150 total anaphase cells were analyzed from n = 3 independent experiments. (**B-D** and **F**) Data represent the mean ± s. e.m. Statistics by one-way ANOVA and Holm-Sidak multiple comparisons test. See also Supplementary Fig. S1.

We measured CIN in tumors from aCSCs versus non-stem cell types [7] by quantifying the frequency of anaphase cell missegregations in H&E-stained sections (Fig. 1A and B). This endpoint allows rigorous assessment of a specific effect on CIN, as defined as continuous chromosomal missegregation, versus aneuploidy, a state of abnormal chromosome number [8,14,15]. Our findings yielded cancer cells displaying several missegregation phenotypes, including anaphase bridges, lagging strands and acentric chromosomes in all tumors examined (Fig. 1A), consistent with CIN representing a common feature of transformed cells [8,15,16]. However, we observed a striking increase in CIN specifically in low-burden aCSC tumors (Fig. 1B) and not in tumors from other cell types (Supplementary Fig. S1A). Importantly, this was not a general property of small versus large tumors as CIN levels were nearly identical when we applied the same criteria across all cell types (Supplementary Fig. S1B). We further observed that CIN decreased in larger, more established aCSC tumors (Fig. 1B), suggesting increased CIN is transient and dissipates as tumors progress. Consistent with this, EpCAM^High^/αvβ3^+^ cells displayed slightly elevated CIN levels, similar to large aCSC tumors (Fig. 1B). Since aCSCs readily differentiate into these cells [7], this supports the possibility that CIN decreases as tumors mature.

We further considered if the increased CIN observed in low-burden aCSC tumors could reflect higher intrinsic levels in these cells. Thus, we measured CIN in freshly sorted HCC38 cells from culture [7] by comparing with a published CIN gene signature [8]. However, this showed that CIN was similar in stem and non-stem cell types (Fig. 1C). We also found no differences in CIN in cell lines belonging to the stem-like claudin-low breast cancer subtype compared to other non-stem subtypes (Fig. 1D). This was performed by comparing the CIN gene signature [8] with published gene sets from 28 breast cancer cell lines previously classified according to their intrinsic subtype [11]. While luminal B cells trended lower, there were no statistical differences with other subtypes (Fig. 1D). We further validated these findings in a subset of cell lines by directly quantifying anaphase missegregations (Fig. 1E and F). Similar to our CIN signature results, we noted reduced levels in luminal B cells, and no differences between basal-like and stem-like claudin-low cells (Fig. 1F). Thus, basal CIN levels alone cannot account for stemness properties. Together these findings suggest that CIN may be induced during tumor initiation, and that aCSCs may possess mechanisms to tolerate the increased stress caused by CIN. Thus, high-CIN may pose a significant barrier to cancer cells during tumor initiation, but one that aCSCs may be uniquely suited to overcome.

### 3.2 Increased c-Jun/JNK stress signaling is a hallmark of aCSCs

**Fig. 2:**
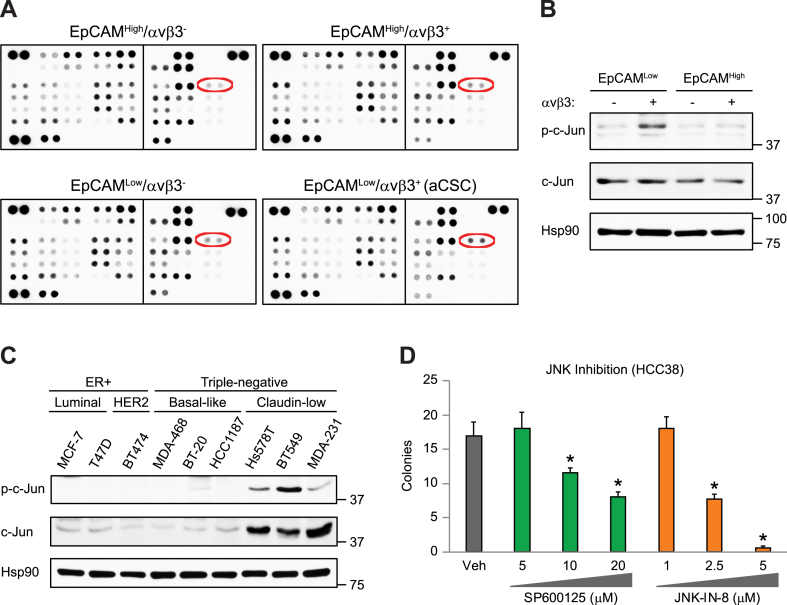
**Enhanced c-Jun/JNK stress signaling in aCSCs.** (**A**) Phospho-array profiling of sorted HCC38 cell types showing enrichment for pS63 c-Jun (red ovals) in aCSCs (EpCAM^Low^/αvβ3^+^). Membranes contain duplicate dots per target phospho-protein. (**B** and **C**) Representative immunoblots confirming enrichment for pS63 c-Jun relative to total c-Jun in sorted aCSCs from HCC38 cells (**B**) as well as stem-like breast cancer cell lines (**C**). Hsp90 is a loading control and molecular weight markers are indicated in kilodaltons. (**D**) HCC38 methylcellulose tumorspheres after treatment with two different JNK kinase inhibitors. Data represent the mean ± s. e.m. Statistics by one-way ANOVA and Tukey's multiple comparisons test. *P < 0.05 relative to vehicle control. (**B-D**) n = 3 independent experiments. See also Supplementary Fig. S2.

To discover critical signaling pathways responsible for CIN tolerance in aCSCs, we performed unbiased phospho-array profiling. Surprisingly, this approach identified pS63 c-Jun as the only major signaling pathway specifically activated in aCSCs (Fig. 2A and B and Supplementary Fig. S2A). We also noted high levels of c-Jun activation in stem-like breast cancer cell lines (Fig. 2C), including cells we previously characterized as surrogates for aCSCs (MDA-MB-231, BT549 and Hs578T) [7]. Enhanced pS63 c-Jun was independent of mutational status as it was observed in stem-like cell lines with distinct oncogenic driver mutations (Supplementary Fig. S2B). While c-Jun expression is essential for cell proliferation and survival as part of the AP-1 transcriptional complex, phosphorylation by c-Jun N-terminal kinases (JNK's) critically regulates the stress response [17,18]. Thus, these new findings are consistent with our prior RNA sequencing results indicating that an enhanced stress response is a unique feature of aCSCs [7]. We further show that c-Jun/JNK stress signaling is required for stemness, since treatment with either of two different JNK inhibitors reduced tumorspheres in HCC38 cells (Fig. 2D). Activation of c-Jun/JNK signaling was previously shown to promote tolerance against CIN-induced stress [[19], [20], [21]], suggesting it may play similar role in aCSCs. In fact, *JUN* is a member of the “Cellular response to stress” gene set we showed was enriched in aCSCs [7], and both *JUN* and *ZEB1* are key genes upregulated in cells with high CIN [8]. Together, our findings identify c-Jun/JNK stress signaling as a major pathway specifically activated in aCSCs that may contribute to CIN tolerance.

### 3.3 AXL is a c-Jun induced gene enriched in aCSCs

**Fig. 3:**
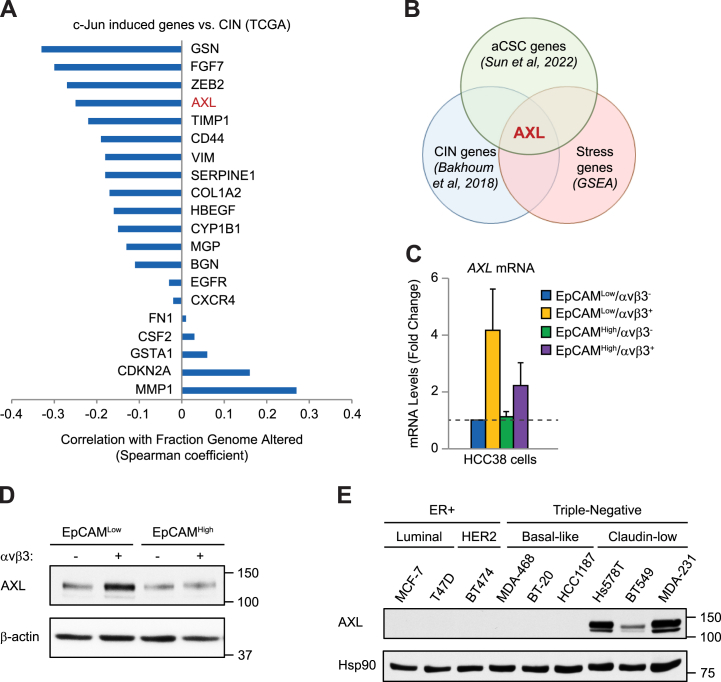
**AXL expression is induced by c-Jun/JNK stress signaling.** (**A**) TCGA analysis comparing established c-Jun transcriptional targets and CIN (fraction of genome altered) in patient breast cancers (invasive carcinoma). Spearman correlation coefficients were determined for each gene using cBioportal and data from the PanCancer Atlas data set (1066 patients). (**B**) Schematic describing the selection criteria for AXL with the indicated gene sets. (**C**) QPCR validation of *AXL* mRNA expression in sorted HCC38 cells types. Samples were run in duplicate with GAPDH as a loading control. Expression is shown relative to the EpCAM^Low^/αvβ3^-^ cells (dashed line). Data represent the mean ± s. e.m. (**D** and **E**) Representative immunoblots for AXL protein expression in sorted HCC38 cells types (**D**) or breast cancer cell lines (**E**). β-actin or Hsp90 are loading controls and molecular weight markers are indicated in kilodaltons. (**C-E**) n = 3 independent experiments. See also Supplementary Fig. S3.

Since c-Jun is part of the AP-1 complex necessary for cell proliferation, and measuring CIN requires cells that are actively transiting through the cell cycle, we reasoned that it would not be possible to assess c-Jun's role in CIN independent of its effects on proliferation. Instead, we decided to identify downstream genes induced by c-Jun that may specifically affect CIN tolerance. For this, we performed TCGA analysis comparing a list of 20 validated genes induced by c-Jun in breast cancers [22] with the “fraction genome altered” as a surrogate endpoint for CIN in patient breast cancers (Fig. 3A). In 15 of the 20 c-Jun induced genes analyzed we observed a negative correlation with CIN (Fig. 3A), including the receptor tyrosine kinase *AXL* (Supplementary Fig. S3A). These findings are consistent with c-Jun acting as a suppressor of CIN [21] and suggest a role for some of these genes as downstream effectors necessary for CIN tolerance. However, only *AXL* was enriched in aCSCs [7], upregulated by CIN [8], and a member of the “Response to Stress” gene set, unique to aCSCs [7] (Fig. 3B). Indeed, *AXL* mRNA and protein were specifically enriched in aCSCs (Fig. 3C and D). We also observed upregulation of AXL protein in stem-like breast cancer cell lines displaying gene expression similarities with aCSCs (Fig. 3E). This includes cells with varying oncogenic driver mutations (Supplementary Fig. S3B), indicating that AXL enrichment is independent of mutational status. Our findings point to enhanced *AXL* expression in aCSCs as a potential CIN tolerance mechanism downstream of activated c-Jun.

### 3.4 AXL is required for CIN-tolerance in aCSCs

**Fig. 4:**
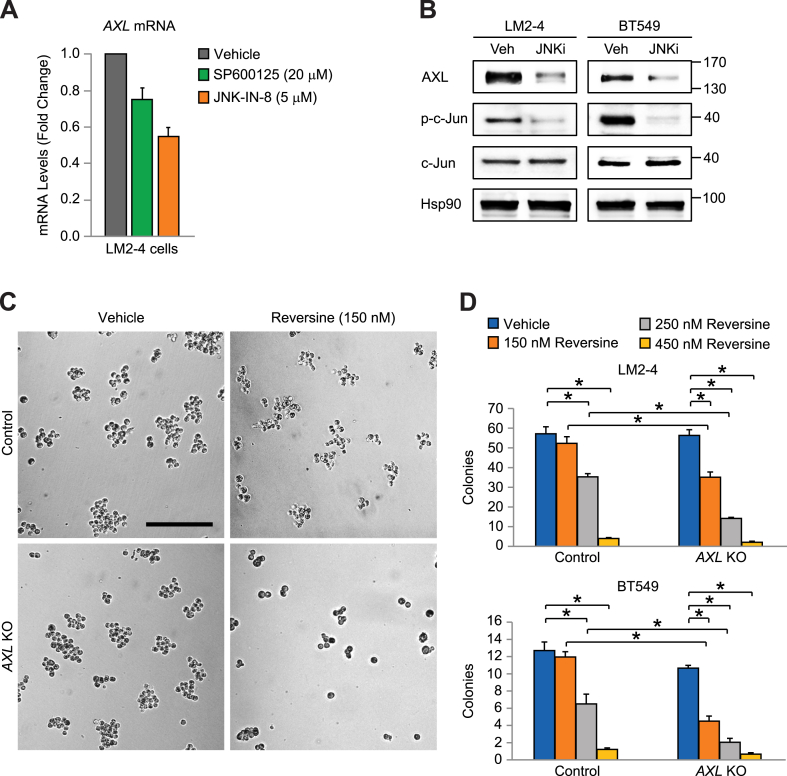
**A c-Jun/AXL signaling module is required for CIN-tolerance in aCSCs.** (**A**) QPCR for *AXL* mRNA after treatment of LM2-4 cells with JNK inhibitors for 24 h. All samples were run in triplicate with β-actin used as a loading control. Data represent the mean ± s. e.m. (**B**) AXL immunoblots of surrogate stem-like cell lines after treatment with DMSO (Veh) or 5 μM of the JNK inhibitor (JNKi) IN-8 for 24 h Hsp90 is a loading control and molecular weight markers are indicated in kilodaltons. (**C** and **D**) Methylcellulose tumorsphere assays with control or AXL KO LM2-4 and BT549 cells treated with Reversine to induce CIN. (**C**) Representative images showing differential response to CIN-induced stress in control and AXL KO LM2-4 cells. Scale bar, 200 μm. (**D**) Statistics by two-way ANOVA and Tukey's multiple comparisons test. *P < 0.05. (**A** and **D**) Data represent the mean ± s. e.m. n = 3 (**A**, **B** and **D**; BT549) or n = 5 (**C** and **D**; LM2-4) independent experiments. See also Supplementary Fig. S4.

To examine a role for c-Jun/AXL as a potential new pathway regulating CIN, we first confirmed that c-Jun activation was required for AXL expression. Indeed, treating cells with the JNK kinase inhibitors SP600125 or JNK–IN–8 significantly reduced *AXL* mRNA (Fig. 4A), while JNK–IN–8 also decreased AXL protein in two different aCSC surrogate cell lines (Fig. 4B). This is consistent with prior characterization of *AXL* as a c-Jun induced gene [23]. To examine how AXL regulates CIN we used CRISPR/Cas9 gene deletion to knockout *AXL* (*AXL* KO) (Supplementary Fig. S4A). Interestingly, *AXL* KO did not affect CIN levels (Supplementary Fig. S4B), suggesting a potential role in tolerating increased CIN, such as that present during tumor progression (Fig. 1B). To mimic the CIN induced in tumors, we used established pharmacological methods of increasing CIN with the monopolar spindle 1 (Mps1) kinase inhibitor Reversine or the Wee1 inhibitor AZD1775 [16]. Treating LM2-4 cells with Reversine or AZD1775 effectively induced CIN by almost 2-fold (Supplementary Figs. S4C and D), consistent with published findings [16], and similar to the increase we observed in low-burden aCSC tumors (Fig. 1B). We then tested if AXL was necessary for tolerating the increased CIN caused by Reversine in methylcellulose tumorsphere assays (Fig. 4C and D). Colony formation in this assay involves many of the same properties required for tumor initiation and thus represents a robust *in vitro* surrogate [[5], [6], [7]]. While low-doses of Reversine had little effect on LM2-4 or BT549 control cells, *AXL* KO cells displayed increased sensitivity, with 150 nM or 250 nM Reversine significantly reducing colony number in both cell lines (Fig. 4C and D). Importantly, *AXL* KO alone had little effect on colony formation. The effects of CIN were more acute on anchorage-independent cells, as the same doses of Reversine produced little response in adherent cell culture (Supplementary Fig. S4E). AXL KO cells were also more sensitive to the CIN induced by AZD1775 (Supplementary Fig. S4F). Overall, these findings point to AXL as an important mediator of CIN tolerance in aCSCs, induced by c-Jun/JNK stress signaling.

## 4 Discussion

CIN is a significant endogenous stress that limits transformation potential [9], but paradoxically also enhances tumor progression by inducing inflammatory signaling [8,16]. Despite this central role in cancer evolution, CIN's relationship with cancer stemness is relatively uncharacterized. Our previous work suggested that regulation of CIN levels may be critical for the aggressive properties of aCSCs. In the present study we now show that CIN levels are upregulated in low-burden aCSC tumors and decrease in more established disease. This suggests that CIN may be induced during early tumor initiation and diminish as tumors grow and differentiate. Such a phenomenon is consistent with the high-CIN noted by others in metastases compared to matched primary tumors [8]. Together, our findings indicate that CIN may represent a critical barrier to tumor initiation that CSCs are uniquely able to overcome.

The stress caused by CIN serves as a significant impediment to transformation in breast epithelial cells [9]. Despite the negative effects on cell survival, many studies characterize CIN as beneficial to tumor evolution by promoting karyotypic heterogeneity [24,25]. Additionally, CIN may activate inflammatory signaling caused by cytoplasmic DNA [8,16], offering an alternative means by which CIN mediates pro-tumorigenic responses. Despite their differences, all of these studies share the premise that high-CIN is pro-tumorigenic, as long as it can be tolerated. Thus, we were surprised to find that intrinsic CIN levels did not vary between stem and non-stem cell types. However, our analysis of the tumors formed from the same cells showed increased CIN specifically in low-burden aCSC tumors. This was not a general property of small versus large tumors, and instead seems to indicate a specific ability of aCSCs to tolerate high-CIN during early stages of tumor initiation. Thus, our findings show that CIN levels alone are insufficient to discriminate between stem and non-stem cell types, and instead support CIN tolerance as a more important attribute of the most aggressive cancer cells.

In this study we characterize a c-Jun/AXL signaling pathway that is critical for CIN tolerance. Unbiased assessment of major signaling pathways activated in aCSCs identified c-Jun/JNK stress signaling as the only pathway significantly upregulated in these cells. While c-Jun expression is essential for cell proliferation and survival as part of the AP-1 transcriptional complex, phosphorylation by JNK's critically regulates the stress response [17,18]. In contrast to prior studies [[19], [20], [21]], we observed no association between CIN levels and pS63 c-Jun, suggesting CIN is not responsible for activating c-Jun. We further identified a new role for AXL as a c-Jun-induced gene in aCSCs that promotes CIN tolerance. In fact, both *JUN* and *AXL* are enriched in aCSCs [7] and cells with high-CIN [8]. Future studies will explore how this new role for AXL may be related to its previously characterized functions in stemness [[26], [27], [28]] and innate immunity [29], including the STING/IL-6 pathway downstream of CIN [8,16]. While AXL also enhances DNA repair [30,31], this is likely not involved since *AXL* gene deletion had no effect on CIN. Together, our new findings highlight the significance of c-Jun/AXL signaling in mediating the response to CIN in aCSCs.

## Author contribution statement

Shahnawaz Baba; Qi Sun: Conceived and designed the experiments, Performed the experiments, Analyzed and interpreted the data. Samson Mugisha: Performed the experiments, Analyzed and interpreted the data. Shreyas Labhsetwar; Richard Klemke: Performed the experiments, Analyzed and interpreted the data, Contributed reagents, materials, analysis tools or data. Jay Desgrosellier: Conceived and designed the experiments, Analyzed and interpreted the data, Wrote the paper.

## Data availability statement

Data is publicly available at:

DOI:10.6075/J0R78FDG.

## Declaration of competing interest

The authors declare that they have no known competing financial interests or personal relationships that could have appeared to influence the work reported in this paper.
