# Supplementary material for: Breast cancer stem cells tolerate chromosomal instability during tumor progression via c-Jun/AXL stress signaling
Source: Heliyon. 2023 Sep 14;9(9):e20182. doi: 10.1016/j.heliyon.2023.e20182 (PMC10559946; doi:10.1016/j.heliyon.2023.e20182)
Supplement: Multimedia component 1 [file mmc1.pdf]

# Supplementary Figure 1

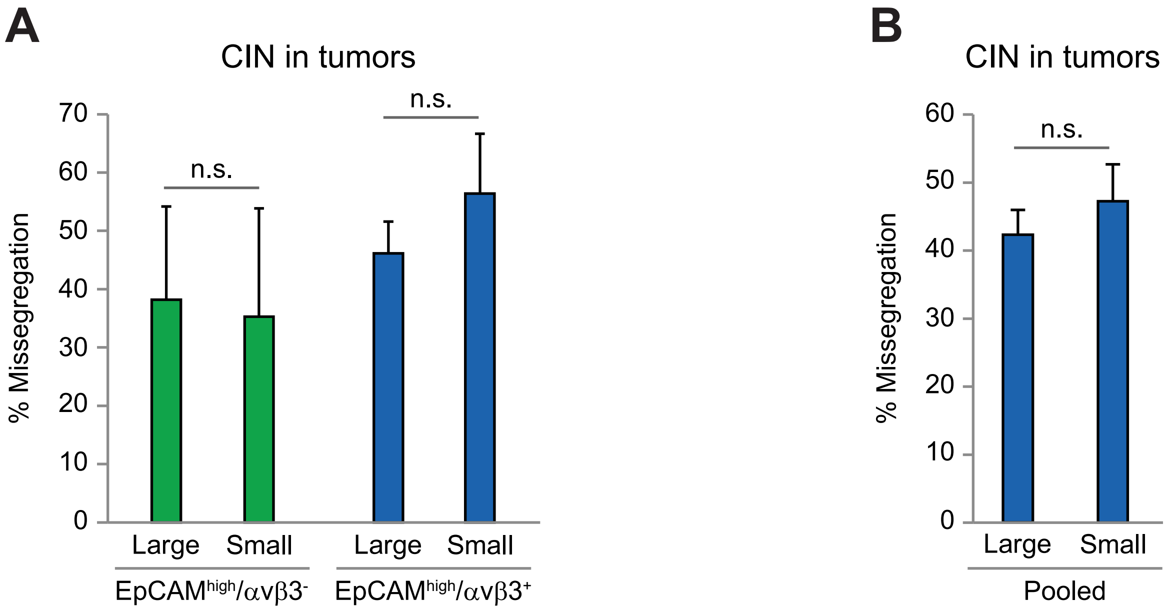


**Supplementary Figure S1. Related to Figure 1.** (**A** and **B**) Quantification of the missegregation frequency in large (>100 $mm^3$) versus small (<100 $mm^3$) tumors formed by sorted HCC38 cells. (**A**) Data is shown separately for $EpCAM^{High}/\alpha v\beta 3^-$ large (n=2) and small (n=2) tumors as well as for $EpCAM^{High}/\alpha v\beta 3^+$ large (n=4) and small (n=2) tumors. No data is shown for $EpCAM^{Low}/\alpha v\beta 3^-$ since all 4 tumors had a volume <100 $mm^3$. (**B**) Pooled data from all sorted cell types, with n=12 tumors in each group. (**A** and **B**) Data shown represent the mean ± s.e.m. from 3 independent experiments. Statistics by two-tailed Student's t-test. n.s.=not significant.

# Supplementary Figure 2

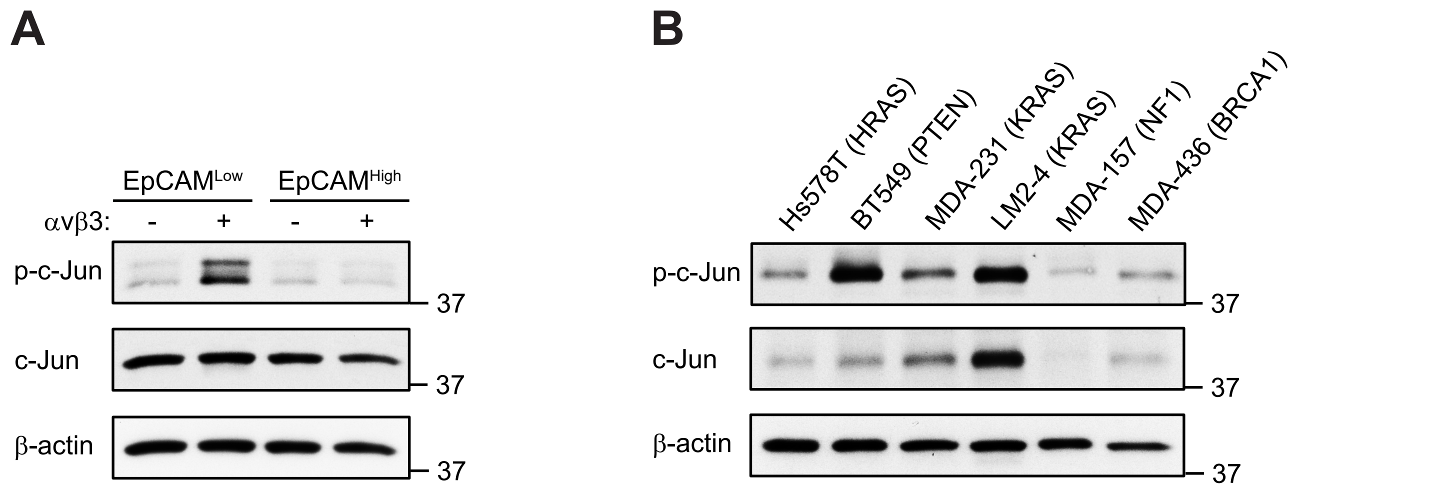


**Supplementary Figure S2. Related to Figure 2.** (**A** and **B**) Representative immunoblots for pS63 c-Jun relative to total c-Jun in sorted aCSCs ($EpCAM^{Low}$/αvβ3) from HCC38 cells (**A**) as well as a panel of claudin-low breast cancer cell lines containing different driver mutations (**B**). β-actin is a loading control and molecular weight markers are indicated in kilodaltons.

# Supplementary Figure 3

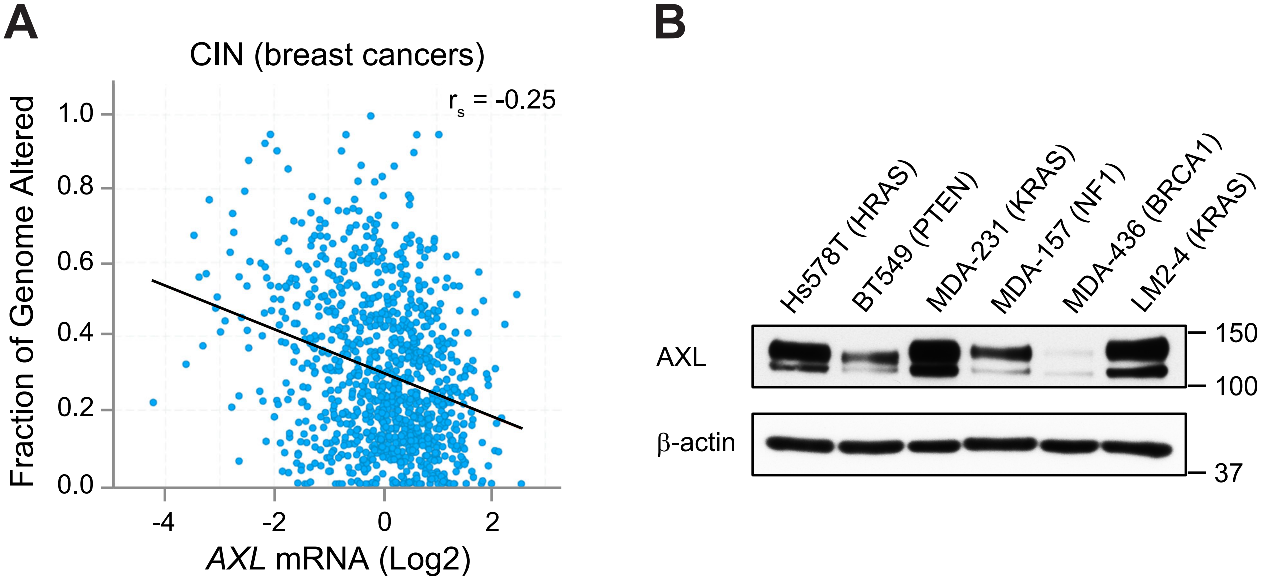


**Supplementary Figure S3. Related to Figure 3.** (**A**) TCGA analysis of patient breast cancers (PanCancer Atlas data set) for *AXL* mRNA expression versus CIN (fraction of genome altered). Statistics by Spearman rank correlation. n=1066 (CIN) or 1007 (mutations) different cancers as indicated by blue dots. (**B**) Representative immunoblot for AXL in a panel of claudin-low breast cancer cell lines harboring different driver mutations. β-actin is a loading control and molecular weight markers are indicated in kilodaltons.

# Supplementary Figure 4

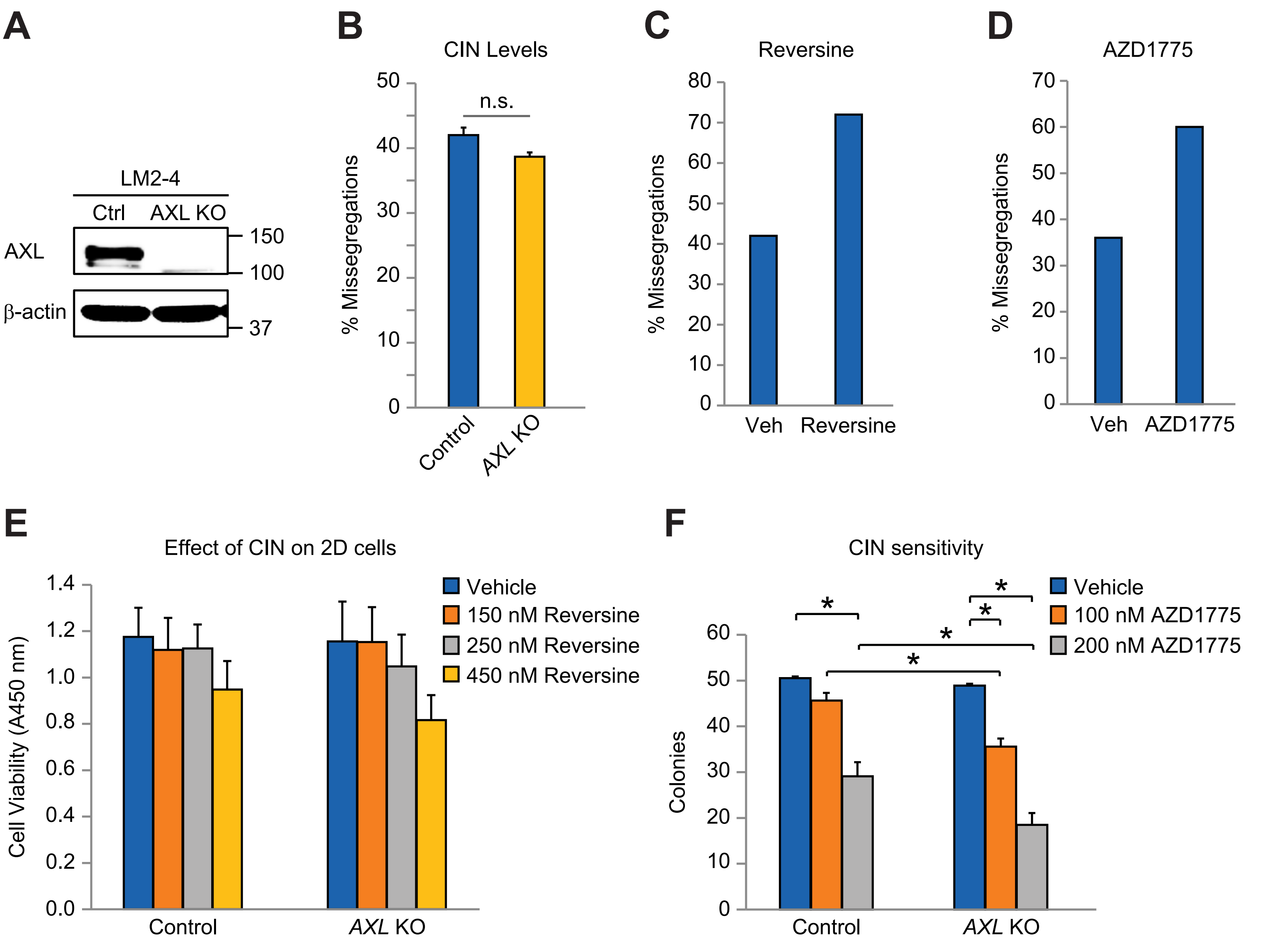


**Supplementary Figure S4. Related to Figure 4.** (**A**) Representative immunoblot for AXL protein levels in LM2-4 cells after CRISPR/Cas9 *AXL* gene deletion. β-actin is a loading control and molecular weight markers are indicated in kilodaltons. (**B**-**D**) Assessment of chromosomal missegregations by DAPI DNA staining. (**B**) Frequency of CIN in control versus AXL KO LM2-4 cells. (**C** and **D**) Representative experiments showing increased CIN levels in LM2-4 cells treated with 250 nM Reversine (14 hr) (**C**) or 500 nM AZD1775 (24 hr) (**D**). (**E**) XTT cell viability assays on control and AXL KO LM2-4 cells grown in 2D culture conditions and treated with the indicated concentrations of Reversine for 48 hr. (**B** and **E**) Statistics by one-way ANOVA and Holm-Sidak multiple comparisons test. n.s.=not significant. (**F**) Methylcellulose tumorsphere assays with control or AXL KO LM2-4 cells treated with the indicated doses of AZD1775 to induce CIN. Statistics by two-way ANOVA and Tukey's multiple comparisons test. **P*<0.05. (**B, E** and **F**) Data represent the mean ± s.e.m. n=3 (**A** and **B**), n=5 (**E**), or n=4 (**F**) independent experiments.

# Supplementary Figure 5

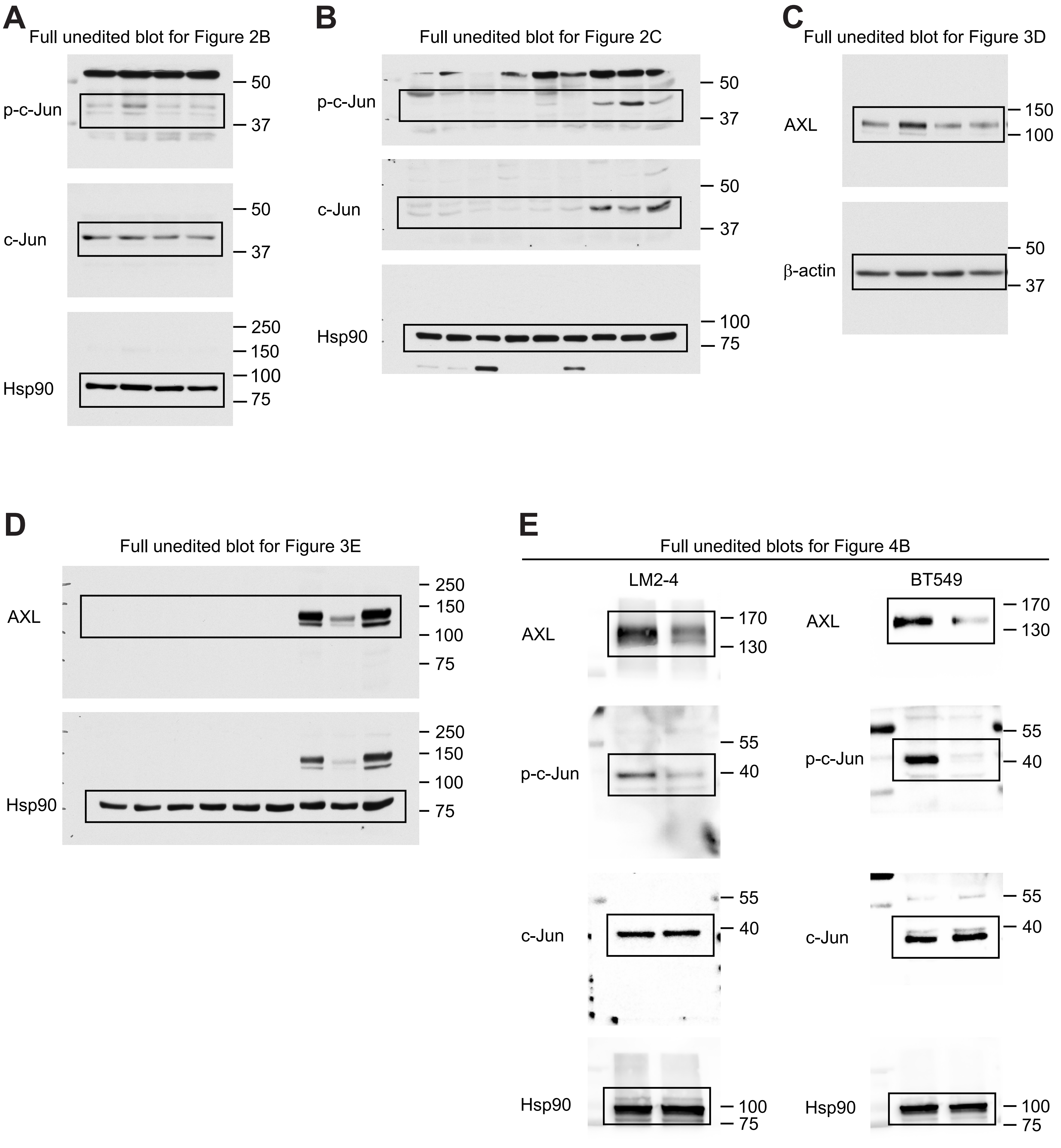


**Supplementary Figure S5. Full unedited Western blots.** Unprocessed scans for the immunoblot data shown in main figures 2B (**A**), 2C (**B**), 3D (**C**), 3E (**D**) and 4B (**E**). Boxes outline the areas cropped for use in figures. Molecular weight markers are indicated in kilodaltons.

# Supplementary Figure 6

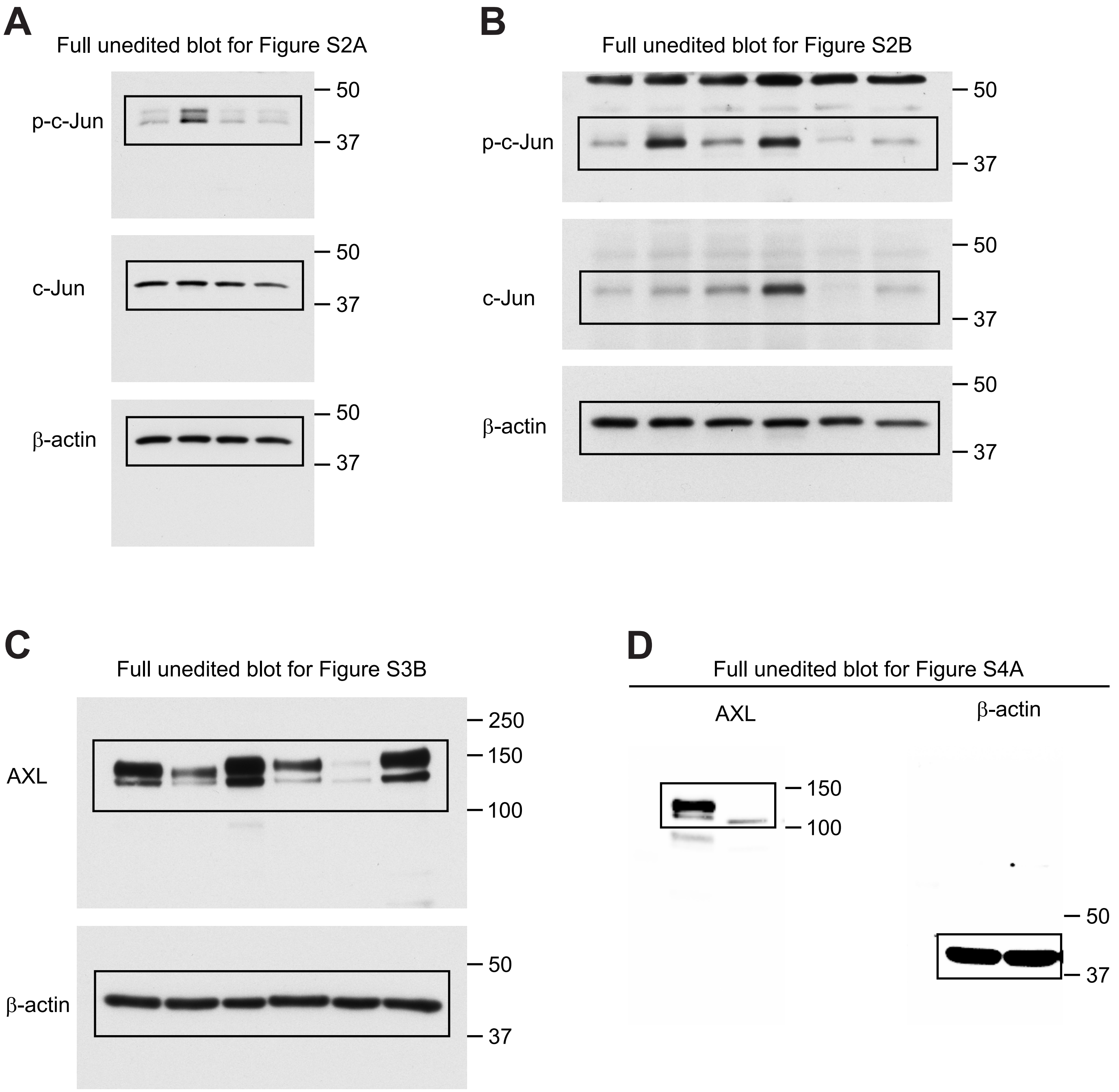


**Supplementary Figure S6. Full unedited Western blots.** Unprocessed scans for the immunoblot data shown in supplementary figures S2A (**A**), S2B (**B**), S3B (**C**), and S4A (**D**). Boxes outline the areas cropped for use in figures. Molecular weight markers are indicated in kilodaltons.
